# The Evolution of Playfulness, Play and Play-Like Phenomena in Relation to Sexual Selection

**DOI:** 10.3389/fpsyg.2022.925842

**Published:** 2022-06-09

**Authors:** Yago Luksevicius Moraes, Jaroslava Varella Valentova, Marco Antonio Correa Varella

**Affiliations:** Department of Experimental Psychology, Institute of Psychology, Universidade de São Paulo, São Paulo, Brazil

**Keywords:** play, gaming, playfulness, sexual selection, mental mechanisms, human evolution, ludicity, evolutionary psychology

## Abstract

By conceptualizing Sexual Selection, Darwin showed a way to analyze intra-specific individual differences within an evolutionary perspective. Interestingly, Sexual Selection is often used to investigate the origins of sports, arts, humor, religion and other phenomena that, in several languages, are simply called “play.” Despite their manifested differences, these phenomena rely on shared psychological processes, including playfulness. Further, in such behaviors there is usually considerable individual variability, including sex differences, and positive relationship with mating success. However, Sexual Selection is rarely applied in the study of play, with exception to what is concerned as infant training behavior for adult sex roles. We offer an integrated grounding of playful phenomena aligning evolutionary propositions based on sexual selection, which might stimulate further exploration of playfulness within evolutionary perspective.

## Introduction

When Darwin (1871/1981) concluded that Sexual Selection explains individual variation in bodily and psychobehavioral traits that could not be explained by differences in habits or survival, he kickstarted promising scientific fields (Cronin, 1993; Miller, 2001). Within a single class of evolutionary processes, Darwin explained the existence of extravagant and costly anatomical/physiological and psychosocial behavioral features not directly related to survival, sex differences across species, ontogenetic differences pre and post puberty, intra-specific individual differences, and the rapid divergence within closely related species (Cronin, 1993; Miller, 2001; Luoto, 2019). This Darwinian approach shed light on many human traits considered evolutionary puzzles, including aspects of play (Chick, 2001; Burghardt, 2005), humor (Greengross and Miller, 2011), sports (Manning and Taylor, 2001), arts, and religion (Zahavi and Zahavi, 1997; Miller, 2001; Varella et al., 2011, 2017; Barash, 2012).

Here we explore how Sexual Selection can foster comprehension about the evolutionary facets of playfulness, which is usually called “play,” but we suggest it is a broader concept not restricted to children. We argue for the benefits of distinguishing play, playfulness and some of the play-like phenomena and suggest how they might relate to Sexual Selection. Furthermore, we present the convergent evidence pointing to the evolved nature of psychological tendencies toward playfulness, focusing on the distinction between play and gaming.

## Definition and Conceptual Distinctions

Depending on epistemological or linguistic differences, “play” can mean either a behavior or a psychological disposition (Sutton-Smith, 2001; Weisfeld and Weisfeld, 2016; Lebed, 2021). Similarly, it can refer to several playful activities. Consequently, “play” and “playfulness” (and sometimes “game”) are used as synonyms. For instance, Huizinga (1938/1980) and Caillois (1958/2001) state that the study of play (or “games” depending on the translation) should include music, theater (role-playing), lottery, philosophy, religion, roller-coaster riding, alcohol drinking, among others. Caillois’ French *jeu* and Huizinga’s Dutch *spel* are neither equivalent to English *play* nor *game*–neither to the Portuguese *brincar* nor *jogar*, respectively (Lebed, 2021). Some languages have only words for a general concept applicable to anything pleasurable, entertaining and autotelic (e.g., Czech, French, German), what we will call “playfulness” henceforward, while other languages make distinctions about different kinds of “play” (e.g., English, Japanese, Portuguese) (Lebed, 2021).

We argue that, beyond linguistic discussions about how narrow or overlapping is their definitions, we suggest using two insights from Evolutionary Psychology: (1) the idea that the human mind has many evolved psychological specializations, (2) the focus on the constellation of underlying psychological capacities being recruited to perform each class of behaviors (Barrett, 2014; Lewis et al., 2017; Pietraszewski and Wertz, 2022). The distinction between the behavioral dimension in the surface and the underlying psychological dimension (cf. Lopes, 2008; Proyer et al., 2021) and the lack of one-to-one correspondence between behavioral and psychological dimensions are crucial. Although many psychological capacities might jointly contribute to each class of behavior, each class of behavior might have their own evolved core capacity/capacities or set of core capacities. For instance, the evolved core capacity for playing might be Playfulness, which is “a propensity to define (or redefine) an activity in an imaginative, non-serious or metaphoric manner so as to enhance intrinsic enjoyment, involvement, and satisfaction” (Glynn and Webster, 1992, p. 85); “the predisposition to frame (or reframe) a situation in such a way as to provide oneself (and possibly others) with amusement, humor, and/or entertainment” (Barnett, 2007, p. 955); “an inclination to pursue activities with the goal of amusement or fun, with an enthusiastic and in-the-moment attitude” (Van Vleet and Feeney, 2015, p. 637); “an individual differences variable that allows people to frame or reframe everyday situations in a way such that they experience them as entertaining, and/or intellectually stimulating, and/or personally interesting” (Proyer, 2017, p. 114).

Since the terms most often associated to “playfulness” are “play” and “game,” Tables 1, 2 overview traits used in the literature to, respectively, define them. Definitions of “play” usually focus on behaviors that occur concomitantly to: intrinsic motivation (e.g., “voluntary,” “autotelic,” “fun”), positive emotions (e.g., “joy.” “happiness,” “pleasure”), and lack of stressors (e.g., “relaxed field,” “play world” or “magic circle”). Authors studying human play define it as “imaginative,” while those focusing on non-humans emphasize functionless-like behavioral modifications. Alternatively, definitions of “game” focus on structured rules; conflicts, there are winners and/or losers, and a quantifiable and important/valued outcome. Therefore, a behavior can be considered “play” when is activated the playfulness capacity which generates a playful state of mind. This playfulness state is usually not explicit in definitions of “game,” and some argue it is not even necessary (e.g., Lebed, 2021), but the rules and outcomes are reframing the conflicts to turn them interesting, and hopefully fun. That means that playfulness can exist even in non-joyful and serious activities (cf. Huizinga, 1938/1980; Caillois, 1958/2001; Suits, 1978; Proyer, 2017). Gaming playfulness may be qualitatively different from playing playfulness, but both are playful.

**TABLE 1:** Traits presented in definitions of “play.”

Trait	Author						
	Avedon and Sutton-Smith (1971)	Burghardt (2005)	Chick (2001)	Eberle (2014)	Gray (2009)	Špinka et al. (2001)	Walther (2003)
Autotelic		X		X	X		X
Behavioral modifications		X	X			X	
Ephemeral	X						X
Have rules					X		X
Imaginative				X	X		X
Lack of stressors		X	X		X	X	
Novelty-seeking				X		X	
Open-ended	X						X
Play-signals			X				X
Positive emotion		X	X	X		X	
Purposeless		X					
Self-handicap						X	
Repetition		X				X	

**TABLE 2:** Traits presented in definitions of “game.”

Trait	Author						
	Avedon and Sutton-Smith (1971)	Juul (2010)	Lever (1978)	Winther-Lindqvist (2019)	Miranda and Stadzisz (2017)	Salen and Zimmerman (2004)	Walther (2003)
Active participation	X	X				X	
Competitive			X	X			
Conflictual	X		X		X	X	X
Clear rules	X	X	X	X	X	X	X
Outcome is emotionally important	X	X		X			
Predefined roles	X		X	X			
Quantifiable outcome		X			X	X	
Unpredicted outcomes	X	X		X	X		

Thus, playfulness can be combined with other traits, as shown by Sutton-Smith’s (2001) analysis of play(fulness) being depicted as: learning (developmental training), a force or drive (fortune-telling, divine providence, and motivation/necessity), a form of dominance (competitions and games of skill), group identification and belonging (festivals and cultural activities), imagination/creativity (arts and make-believe), self -expression and individualization (hobbies and recreation), and as subversion (jokes and sarcasm). The combination of Playfulness with the tendency for physical activity (i.e., a voluntary movement with energy costs superior to rest levels, Caspersen et al., 1985) might give rise to the class of behavior known as play (cf. Špinka et al., 2001; Burghardt, 2005). Thus, the different subtypes of play (e.g., with object, rough and tumble, roleplaying, rule based) would be further combinations with other psychological capacities (e.g., manipulation, ritualized competition, imagination/representation, normativity). Another example of evolved features in the human mind is artisticality, the autotelic pleasurable capacity for extraordinary ornamentation/aesthetic improvements (Varella et al., 2017; Varella, 2021). The combination between the tendency for physical activity and artisticality gives rise to the different artistic modalities.

Recognizing that behaviors recruit multiple psychological components enables a better understanding of overlapping cases and why many cultures use “play” across different behavioral classes. From a combination of a few psychological invariants one can observe a broad behavioral diversity and overlap of classes. Recruitment of language with playfulness may lead to play on words, jokes, celebrity voice impersonations, or word games, such as crosswords, hangman game, or Wordle. Recruitment of capacities for narrative, exploration, normativity and playfulness may lead to role-playing games (RPGs). Recruitment of language/narrative and artisticality leads to poetry and literary arts. Recruitment of playfulness and artisticality results in colorful domino art, Pictionary, musical jokes and parodies, circus arts, or hobby artists. Recruitment of ritualized competition/cooperation, normativity, self-overcoming and playfulness leads to most sportive games, chase tags, and tabletop games. Recruitment of ritualized competition/cooperation, normativity, self-overcoming and artisticality results in rhythmic gymnastics, figure skating, synchronized swimming, slam dunk contest, keepie-uppie competition, dancing contest, capoeira, or rap battles if language is also recruited.

Furthermore, if we consider that most humans are not serious professional sportspeople, gamers, or artists (Moraes, 2021), but nevertheless recruit the universal capacities underlying those tendencies in their leisure time, hobbies and self-entertainment moments, the contribution of playfulness becomes clear and ubiquitous even in adulthood. About 22% individuals consider free-time a time to fun and about 13% consider it a time to dedicate to one’s own hobby (Mingo and Montecolle, 2014). Playfulness may lead to many pleasurable activities that can or cannot latter become professionalized.

## Play and Games as Stemming From Evolved Propensities

The psychological tendency toward play exhibits many criteria of evolved adaptation. It is present in all human cultures (Huizinga, 1938/1980; Gosso and Otta, 2003; Sandseter and Kennair, 2011) and is typical of mammals (Špinka et al., 2001; Burghardt, 2005), especially regarding large-brained mammal orders (Iwaniuk et al., 2001), suggesting play is at least as antique as the first mammals (Late Triassic), but it might have independently evolved in birds with delayed reproduction (Diamond and Bond, 2003; Kaplan, 2020), fish, turtles and octopuses (Kuba et al., 2003; Burghardt, 2005). Playful phenomena are culturally valued (Sutton-Smith, 2001). Play also has high costs (Harcourt, 1991; Greve et al., 2014; Froehle et al., 2019), is observable very early in ontogeny (Eibl-Eibesfeldt, 2017; Winther-Lindqvist, 2019), provides physical, cognitive and social benefits (Špinka et al., 2001; Bjorklund and Pellegrini, 2002; Sandseter and Kennair, 2011). It results in specific emotional states (Špinka et al., 2001; Burghardt, 2005; Davis and Panksepp, 2011), has a heritable component (in mice, Walker and Byers, 1991; in humans, Olson et al., 2001), and it is at least partly modular, since its content may change, but it does not disappear under any known disorders, regardless if it is an intellectual, personality, endocrinal or mood disorder (cf. Berenbaum and Hines, 1992; Davis and Panksepp, 2011; Papoudi and Kossyvaki, 2019).

Gaming is considered exclusively human (Breuer et al., 2019), arguably because we usually require some spoken acceptance of explicit rules. Not all animals use violence in competitions, but some of them have non-violent ritualized competitions (Maynard Smith, 1974) that could be called “games” if there was any evidence of mutual agreement. Even rough-and-tumble play and play fighting have some recognizable rules, like controlling own force to avoid harming the play partner, signals of “this is play” (“play face” in primates) and role-reversal (Chick, 2001; Špinka et al., 2001; Burghardt, 2005). Games have also existed for at least 5 or 6 thousand years (Rollefson, 1992), but considering they are universal (Roberts et al., 1959; Chick, 1998; Voogt, 2017), they might have existed for much longer. Indeed, anthropologists have suggested that some archeological artifacts originally considered “works of art” or “religious tools” may be pieces of unknown games (Culin, 1907/2007; Avedon and Sutton-Smith, 1971). Furthermore, gaming is culturally valued (Avedon and Sutton-Smith, 1971; Apostolou et al., 2014; Crist et al., 2016), is associated with physical, cognitive and social benefits (Caillois, 1958/2001; Roberts et al., 1959; Zimmer, 1987; Kaufman et al., 2019). It includes specific mental states (Walther, 2003; McGonical, 2011), and is costly due to self-handicapping rules (Suits, 1978). Gaming develops later than play (Winther-Lindqvist, 2019), and makes individual differences more evident (Caillois, 1958/2001). At least sportive games also have a heritable component (Olson et al., 2001; Tucker and Collins, 2012).

## Play as a Result of Natural and Sexual Selection

Different evolutionary mechanisms can explain different ludic phenomena (cf. Liebold et al., 2019). Play behavior is usually seen as a result of Natural Selection, a way for training hard-to-master skills that would require dangerous conditions if play did not existed (Špinka et al., 2001; Bjorklund and Pellegrini, 2002; Burghardt, 2005). Meanwhile, Sexual Selection is used to explain the evolutionary functions of arts (e.g., Varella et al., 2011, 2017), sports (e.g., Lombardo, 2012; Deaner et al., 2016) and even playfulness as an individual difference trait (e.g., Chick, 2001; Moraes et al., 2021). Play’s costs imply play can be a reliable signal of health, and consequently, this might be generalizable to adulthood. The *Signal Theory of Playfulness* (Chick, 2001) postulates a playful attitude not only signals a healthy condition, but other desirable trait, like non-aggressiveness in males and youthfulness in females. Contrarily, theories about the origins of sports, generaly understood as playful competitions of physical skills, postulates sport play signs fighting and hunting skills in controlled conditions to attract mates (intersexual competition), coalitional allies, status and/or resources (intrasexual competition; see Deaner et al., 2016). Moraes et al. (2021) found that in men other-directed playfulness positively predicted the number of long-term and short-term partners, while in women whimsical playfulness positively predicted the number of short-term relationships. Thus, playfulness is possibly being sexually selected in both sexes because playful adults have more partners. Sexual selection also might explain why the adult play (gaming) is, apparently, human-only and why there are robust cross-cultural sex differences (Roberts et al., 1959; Gray, 2004; Deaner and Smith, 2013; Moraes, 2021).

Sexual Selection shapes characteristics that tend to be costly, sometimes even reducing the chances of survival, are highly variable within the same species, they frequently develop in only one of the sexes and/or after puberty, and are species-specific (Zahavi and Zahavi, 1997; Miller, 2001; De Block and Dewitte, 2007; Lange et al., 2019; Petrie, 2021). Sexual Selection can also be roughly divided into Intersexual Selection, which involves adaptations to choose or be chosen as a mate by an opposite-sex individual, and Intrasexual Selection, which involves adaptations to defeat or intimidate same-sex individuals (Apostolou, 2015; Lange et al., 2019; Petrie, 2021).

More playful individuals are preferred as mates (Chick et al., 2012; Proyer and Wagner, 2015), and playfulness is also positively correlated with physical fitness (Proyer et al., 2018), relationship satisfaction (Brauer et al., 2021) and is subject to assortative mating (Chick et al., 2020), which can explain its heritability. Additionally, players of sportive games have more sexual partners (Faurie et al., 2004). However, there is almost no studies about the generalizability to other games and a few exceptions found mixed results (Lange and Schwab, 2019; Moraes, 2021). If neither Natural Selection, nor Intersexual Selection were alone responsible for the evolution of gaming, Intrasexual Selection might also play some role (cf. De Block and Dewitte, 2007). Thus, gaming may be a way to compete for resources that will indirectly increase the fitness, such as status or coalition allies (De Block and Dewitte, 2007, 2009; Balish et al., 2016; Winegard et al., 2018). Importantly, these selective forces may act simultaneously in different degrees. De Block and Dewitte (2009) argue there are so many sportive games because sports act as honest signals of good genes, which must be informative (provide evolutionary relevant information), accurate (hard to falsify, reliable) and transparent (spectators should be able to decode the sign). But there is often a trade-off among these three qualities and different sports target different proportions. Furthermore, there might be redundant and multiple signals display within ornamental signaling (Valentova et al., 2017; Pereira et al., 2019). Additionally, sports that lost their signal value to new ones can continue through exaptation, i.e., acquiring new functions, like when fans try to earn money through bets or try to impress people displaying their knowledge about game-related history and statistics, but the original (distal) function of the sport was to signal players’ physical skills.

Some attempts to apply an evolutionary perspective in the study of non-sportive games include Gray’s (2004) analysis, showing that men play casino games more often than women as a byproduct of male general higher risk-taking and dependency on resource holding to attract mates. Some researchers study digital games from an evolutionary framework (e.g., Mendenhall et al., 2010; Lange et al., 2019), but it is often not clear if they talk about *digital gaming* or *digital pretense play*. We have no knowledge of attempts to explain the adaptive values of other games through evolutionary mechanisms, but it would surely be interesting. For instance, card games rely on deception and social manipulation (Altice, 2014) and, consequently, they may be the products of sexual selection processes combining playfulness, cheating and cheater-detection behavioral and mind reading (cf. Varella, 2018).

## Discussion

An evolutionary framework has greatly contributed to the study of play, but it has been focused on “repeated, incompletely functional behavior differing from more serious versions structurally, contextually, or ontogenetically, and initiated voluntarily when the animal is in a relaxed or low-stress setting” (Burghardt, 2005, p. 51). Though it captures what is usually thought when someone says “play,” it does not capture all the playful experiences one can get, nor capture the proper psychological level in which evolution operates (Barrett, 2014). Phenomena as “game,” “sport,” “music-playing,” “role-playing,” and “fortune-telling” are called “play” in some languages and not in others (Huizinga, 1938/1980). This might reflect a phylogenetic continuity or that similar combinations of psychological mechanisms are being used. We suggest that all of them use in some level the psychological capacity of playfulness. They may have evolved through Natural Selection as mechanisms to actively search for ways of increasing one’s own fitness, but also Sexual Selection acted on how they are used to create complex phenomena as gaming and arts (Liebold et al., 2019).

We argue that there is enough robust evidence to support the existence of these mechanisms (Caillois, 1958/2001; Scott and Godbey, 1992; Walther, 2003; Conway, 2010; Argento et al., 2017; Zosh et al., 2018; Lebed, 2021). What is needed is a good theory to avoid the “theory crisis” (Eronen and Bringmann, 2021). Sexual Selection might be this theory. However, researchers should accept some basic principles which make studies richer and less prone to appear contradictory, and avoid misunderstandings (Varella et al., 2013). Some of these principles are already used in other areas, like bio-musicology (Fitch, 2015): recognize that these behavioral phenomena are composed of many interacting psychological components; look for cross-cultural and inter-specific homologies and analogies; avoid elitism and focus on ecological validity; consider the Tinbergen’s questions–mechanism, ontogeny, function, phylogeny, plus their updates (e.g., subjective experience, Burghardt, 2005; and sociocultural history as medial explanation between proximal and distal ones, Varella et al., 2012).

Importantly, Darwin’s (1871/1981) theory has since been expanded and updated. Different mechanisms of Sexual Selection have been proposed (cf. Puts, 2010; Shuker and Kvarnemo, 2021). For instance, men may play sports more often because women prefer sportive men (Faurie et al., 2004), because sports work as behavioral armaments in intrasexual competition (Lombardo, 2012) or as displays for potential parents-in-law (Apostolou, 2017), besides other bio-socio-cultural functions. Psychological traits can function as both armaments and ornaments (Berglund et al., 1996).

Here we take this sesquicentennial celebration as an opportunity to invite the researchers to get inspired by Darwin’s pioneering and courageous move of looking into intra-specific differences and searching the mechanisms that drive them, in order to disentangle some of the most intriguing aspects of human life: How do playful phenomena (games, sports, arts, religion, humor, flirting, etc.) differ among each other? And how they can contribute to individual survival and reproductive success? Future studies should consider the overlapping among playful phenomena, their multiple levels, cultural meaning, interspecies similarities, organism’s developmental stage and both Natural and Sexual Selection.

## Author Contributions

YM: idealization and writing of the manuscript. JV: supervision and revision of the manuscript. MV: manuscript writing and revisions. All authors contributed to the article and approved the submitted version.

## Conflict of Interest

The authors declare that the research was conducted in the absence of any commercial or financial relationships that could be construed as a potential conflict of interest.

## Publisher’s Note

All claims expressed in this article are solely those of the authors and do not necessarily represent those of their affiliated organizations, or those of the publisher, the editors and the reviewers. Any product that may be evaluated in this article, or claim that may be made by its manufacturer, is not guaranteed or endorsed by the publisher.
